# Lenvatinib plus transarterial chemoembolization with or without immune checkpoint inhibitors for unresectable hepatocellular carcinoma: A review

**DOI:** 10.3389/fonc.2022.980214

**Published:** 2022-09-28

**Authors:** Liwei Sun, Xuelong Xu, Fanguang Meng, Qian Liu, Hankang Wang, Xiaodong Li, Guijie Li, Feng Chen

**Affiliations:** ^1^ Department of Radiology, The First Affiliated Hospital of Shandong First Medical University & Shandong Provincial Qianfoshan Hospital, Shandong Medicine and Health Key Laboratory of Abdominal Medicine Imaging, Jinan, China; ^2^ Graduate school, Shandong First Medical University & Shandong Academy of Medical Sciences, Jinan, China; ^3^ Zibo Maternal and Child Health Care Hospital, Zibo, China; ^4^ Department of Radiology, The Second Affiliated Hospital of Zhejiang University School of Medicine, Zhejiang, China

**Keywords:** lenvatinib, transarterial chemoembolization, immune checkpoint programmed death factor 1 inhibitors, immune checkpoint inhibitors, unresectable hepatocellular carcinoma

## Abstract

Lenvatinib plus transarterial chemoembolization (TACE)have become the first choice for patients with hepatocellular carcinoma (HCC) that are unsuitable for TACE. Sorafenib plus TACE therapy for patients with portal vein tumor thrombus (PVTT) achieved positive results. However, Lenvatinib plus TACE appeared to achieve a more advantageous result for these patients based on the phase 3 REFLECT trial. Both TACE and lenvatinib therapy have immune-stimulating effects, so would lenvatinib plus TACE and immune checkpoint inhibitors be an advantageous therapy for unresectable HCC (uHCC)? Thirteen articles from PubMed were explored to determine the efficacy and safety of lenvatinib plus TACE with or without PD-1 inhibitors therapy. Most of the adverse events (AEs) were manageable. Lenvatinib plus TACE therapy was superior to lenvatinib monotherapy with intermediate stage HCC especially beyond up-to-seven criterion and was superior to TACE monotherapy in patients with uHCC or sorafenib plus TACE therapy in patients with PVTT. Objective response rates (ORRs) of 53.1%–75%, median progression free survival (PFS) of 6.15–11.6 months, and median overall survival (OS) of 14.5–18.97 months were achieved in the lenvatinib plus TACE group. Levatinib plus TACE and PD-1 inhibitors achieved ORRs of 46.7% –80.6%, median PFS of 7.3–13.3 months, and median OS of 16.9–24 months. Control studies also confirmed the triple therapy was superior to lenvatinib plus TACE in patients with uHCC. Overall, the triple therapy is a promising treatment for patients with uHCC, including main PVTT and extrahepatic metastasis. Lenvatinib plus TACE therapy was also preferable for intermediate stage HCC beyond up-to-seven criterion and for patients with PVTT.

## Introduction

Hepatocellular carcinoma (HCC) is malignant, and the median overall survival (OS) of HCC with Barcelona clinic liver cancer (BCLC) 0/A, B, C, and D was longer than 5 years, 2.5 years, 2 years and 3 months, respectively (1). Additionally, the poorer the liver function, the higher the incidence of treatment-related poisoning events (2–5). Transarterial chemoembolization (TACE) was the first choice for patients at the intermediate stage (BCLC stage B). However, repeated TACE treatment could decrease the liver function and cause failure to accept follow-up systematic treatment. The increased times of TACE treatment resulted in the decline in the response rate of tumor tissue to treatment (6–9). The phenomenon was known as TACE refractoriness/TACE failure, which was defined by the Japan Society of Hepatology in 2010 (7). What occurs if systemic therapy is applied before TACE treatment? The results of a prospective study of lenvatinib as initial treatment in HCC at BCLC substage B2 showed the median OS and progression free survival (PFS) were 17.0 and 10.4 months, and objective response rate(ORR)was 70.0%, respectively (10). The albumin-bilirubin (ALBI)score was sustained in the lenvatinib group, whereas it declined in the TACE group after the treatment (8). Kudo et al. also published an article explaining that HCC at BCLC stage B, especially HCC with multiple heterogeneous nodules, need lenvatinib pretreatment before TACE to achieve a high tumor response rate, preserve liver function, and prolong PFS and OS (11–13). The first choice of the treatment of intermediate HCC with a high tumor burden, especially beyond the up-to-seven criterion, is no longer TACE (11, 14–17). The intermediate stage of HCC sank and became multifocal, with preserved liver function, and had considerable heterogeneity: from the Child-Pugh score 5 to Child-Pugh score 9, tumor size from ≥5 cm to >10 cm, and the number of nodules from 4 to >10 (1, 11, 18–20). According to the 2022 BCLC version stratifications, TACE is suitable for HCC with well-defined nodules, preserved portal flow, and selective access. Systemic therapy is suitable for the BCLC stage B HCC that are diffuse and infiltrative, with extensive liver involvement, but there was no clear dividing line between the two (1, 21). HCC in an advanced-stage (BCLC stage C) with vascular invasion or extrahepatic spread, ECOG PS ≤ 2, and preserved liver function should be evaluated for systemic therapy (1). The combination of atezolizumab with bevacizumab is the first-line treatment, exhibiting a breakthrough ORR of 33.2% and median PFS of 6.8 months and proving the superiority compared to sorafenib in survival benefit (22–24). According to Maesaka et al., although the median PFS was significantly longer in the atezolizumab plus bevacizumab group (8.8 months vs. 5.2 months), there were no significant differences in terms of median OS (not reached vs. 20.6 months) or ORR (43.8% vs. 52.4%) (25). If lenvatinib plus TACE therapy can improve PFS, and achieve results matching the atezolizumab plus bevacizumab therapy requires further study.

The TACTICS trial confirmed the advantage of sorafenib plus TACE compared to TACE alone for patients with unresectable hepatocellular carcinoma (uHCC) (18 at BCLC stage C), with a better PFS (25.2 vs 13.5months) and a higher ORR (71.3% vs 61.8%) (26). According to the phase 3 REFLECT trial, the OS of lenvatinib and sorafenib was 13.6 and 12.3 months, and patients in the lenvatinib group exhibited a longer median time to progression (TTP) compared with sorafenib (8.9 vs. 3.7 months), a higher ORR (21.4% vs. 9.2%), and a longer PFS (7.2 vs. 4.6 months) (8, 13). However, there was no comparison between lenvatinib plus TACE therapy and sorafenib plus TACE therapy. Patients with liver occupation greater than 50%, bile duct invasion, a Child-Pugh class B, or main portal vein tumor thrombus (PVTT) were excluded from the phase 3 REFLECT trial (13). We need to verify the efficacy of these two tyrosine kinase inhibitors (TKIs) plus TACE treatments at various tumor stages.

The occurrence and progression of HCC are based on the inflammatory environment of the liver. Many immune related factors or cells provide a immunosuppression tumor microenvironment (TME) for tumor cells (5, 27). Immune checkpoint inhibitors (ICIs) is a research hotspots, including the inhibition of immune checkpoint programmed death factor 1 (PD-1), programmed death factor ligand 1 (PD-L1), and cytotoxic T-lymphocyte-associated protein 4 (CTLA-4) (5). PD-1, a transmembrane receptor, is expressed by activated T cells, B cells, natural killer cells, and antigen-presenting cells. PD-L1 is expressed by cancer cells, which would combine with PD-1 and escape from immunosurveillance (5, 27–29). The inhibition of these two targets destroy the immunosuppression TME of tumors. However, approximately two-thirds of HCC did not respond to immunotherapy alone, which illustrated the complex interaction of multiple immunosuppressive mechanisms in the TME. More optimized treatment strategies need to be formulated, and combination therapy is the first choice (30). The combination therapy of lenvatinib plus pembrolizumab produced an ORR of 46% and a median OS of 22.0 months, according to a Phase Ib Study. The FDA approved lenvatinib plus pembrolizumab as a first-line treatment for uHCC that is not amenable to locoregional therapy (31–33). This indicated that lenvatinib had synergistic effects with PD-1 inhibitors. Whether lenvatinib plus TACE combined with PD-1 inhibitors, such as pembrolizumab, is better than lenvatinib plus TACE therapy and what synergistic effects exist among TACE, lenvatinib, and PD-1 inhibitors are also the key points to be discussed in this review.

Based on the above, this review embodied 13 studies by the end of April 2022 through PubMed to explore the advantages of lenvatinib plus TACE therapy and lenvatinib plus TACE and PD-1 inhibitors in patients with uHCC.

## Therapeutic responses of lenvatinib plus TACE therapy versus lenvatinib or TACE monotherapy

First, we determine whether lenvatinib plus TACE therapy has advantages over TACE or lenvatinib monotherapy. This chapter covers 3 articles comparing lenvatinib plus TACE therapy with TACE monotherapy for patients with uHCC, lenvatinib monotherapy for intermediate HCC mostly beyond up-to-seven criterion, and one demonstrated the therapeutic effect of lenvatinib plus TACE on uHCC with PVTT, in a total of 218 people (2, 17, 34, 35).

### Objective response rate

Four studies reported response assessments based on the modified Response Evaluation Criteria in Solid Tumors (mRECIST), classified as complete response (CR), partial response (PR), stable disease (SD), and progression of disease (PD). The ORR (CR + PR) was reported in three studies, and the outcomes are shown in **Table 1**. ORR of the lenvatinib plus TACE group vs. the lenvatinib-alone group was 63.2% vs. 63.2%, p = 1.0, respectively. However, CR was 15.8% in the lenvatinib plus TACE group and was 10.5% in the lenvatinib-alone group (17). ORR of the lenvatinib plus TACE group vs. TACE-alone group was 68.3% vs. 31.7%, p<0.001 (34). Chen et al. reported that the ORR of the lenvatinib plus TACE group was 75%, significantly better than the phase 3 REFLECT trial, even when including 25% HCC with main PVTT (35).

**Table 1:** Lenvatinib plus TACE therapy compared with lenvatinib or TACE monotherapy.

Author/Reference numbers	Year	Country	Treatment (number of patients)	Follow-up time	Median OS/OS rate	Median PFS/PFS rate	TTP	ORR	Main characteristics of patients
Shigeo Shimose (2)	2021	Japan	Lenvatinib+AT (24) or lenvatinib (24)	NA	not reached vs. 16.3 months	NA	NA	NA	BCLC stage B (100%); Beyond up-to-seven criteria (87.5% vs. 91.6%); ALBI grade 2 (54.17% vs. 66.67%)
Yuwa Ando (17)	2021	Japan	Lenvatinib+TACE (19) vs lenvatinib (19)	14.8 vs. 14.3 months	not reached vs. 16.9 months	11.6 vs. 10.1 months	NA	63.2% vs. 63.2%	BCLC stage B (100%); Beyond up-to-seven criteria (68.4% vs. 63.16%); ALBI grade 2 (31.58% vs. 31.58%)
Zhigang Fu (34)	2021	China	Lenvatinib+TACE (60)/TACE (60)	11.6 vs. 17.5 months	The 1-year and 2-year OS rates were 88.4% and 79.8% vs. 79.2% and 49.2%	The 1-year and 2-year PFS rates were 78.4% and 45.5% vs. 64.7% and 38.0%	NA	68.3% vs. 31.7%	Child-Pugh grade B (6.7% vs. 5.0%); PVTT (35.0% vs. 45.0%); Extrahepatic spread (15.0% vs. 15.0%); AFP≥400(45.0% vs. 45%); BCLC stage A (3.3% vs. 5.0%)/B (55.0%vs43.3%)/C (41.7% vs. 51.7%); ALBI grade 2-3 (68.33% vs. 73.33%)
Ruiqing Chen (35)	2022	China	Lenvatinib+TACE (12)	15.2 months	16.9 months	6.15 months	NA	75%	PVTT type II (75.0%), III (25.0%); Extrahepatic spread (58.3%); BCLC stage C(100%)

OS, overall survive; PFS, progression-free survival; TTP, time to progression; ORR, objective response rate; AT, trans-arterial therapy; NA, not available; BCLC, Barcelona clinic liver cancer; ALBI, albumin-bilirubin; TACE, transarterial chemoembolization; PVTT, portal vein tumor thrombus; AFP, alpha-fetoprotein.

### Progression free survival and overall survival

Ando et al. reported that the median PFS of the lenvatinib plus TACE group vs. lenvatinib-alone group was 11.6 vs. 10.1 months, p = 0.019, respectively. Child-Pugh score 5 and lenvatinib followed by TACE were the predictive factors of PFS in a multivariate analysis (17). A prospective study also showed that the Child-Pugh score was a momentous factor for the prognosis (10). The 1-year and 2-year PFS rates were 78.4% and 45.5% vs. 64.7% and 38.0%, p<0.001, in the TACE plus lenvatinib group vs. TACE alone, respectively (34). Chen et al. reported that the median PFS of lenvatinib plus TACE group was 6.15 months (35). Shimose et al. and Ando et al. reported that the median OS of the lenvatinib plus TACE group vs. lenvatinib-alone group were not reached vs. 16.3 months, P = 0.01 and not reached vs. 16.9 months, p = 0.007, respectively (2, 17). The independent predictive factors of OS were transarterial therapy and ALBI grade 1 according to Shimose et al. (2). Child-Pugh score 5, serum AFP level <400 ng/mL, and lenvatinib followed by TACE were the independent predictive factors of a longer OS in multivariate analysis according to Ando et al. (17). According to Yao et al., a high level of AFP was correlated with poor prognosis in HCC. This was possibly because it was positively correlated with the weakening of the immune stimulation effect of dendritic cells (DCs) on T cells (36). The 1-year and 2-year OS rates were 88.4% and 79.8% vs. 79.2% and 49.2%, p=0.047, in the TACE plus lenvatinib group vs. TACE monotherapy, respectively (34). A treatment option was identified as an independent prognostic factor for OS in the multivariate analysis and the benefits of the total population were consistent with BCLC stage B and C in the lenvatinib plus TACE group (34). The relative dose intensity (RDI) was relevant to the therapeutic response of lenvatinib, including the PFS and OS (8).Chen et al. reported the median OS of the lenvatinib plus TACE group was 16.9 months (35). This is slightly lower than the results published by Shimose et al. and Ando et al. However, the patients included in Shimose et al. and Ando et al. had HCC at BCLC stage B, whereas Chen et al. included patients with HCC and PVTT (2, 17, 35).**Table 1** shows the details.

### Change of liver function and adverse events

ALBI grade was an important factor associated with survival in patients with HCC (37, 38). According to Shimose et al., age and ALBI were the first and second splitting variables for arterial therapy (AT), respectively (2). The median ALBI score in the TACE plus lenvatinib group before TACE, and 1 and 2 months after TACE, and at the end of treatment was −2.52, −2.48, −2.51, and −2.44, respectively, and there was no significant difference (17). According to Fu et al., there was no dramatical change in the Child-Pugh score between the baseline and the first follow-up after treatment in the TACE plus lenvatinib group and TACE group (34). Common adverse events (AEs) included hypertension, hemorrhage of the digestive tract, liver dysfunction, ascites, proteinuria, fatigue, anorexia, hand-foot skin reaction (HFSR), hypothyroidism, diarrhea, and hoarseness. There was no obvious difference in AEs between the groups. Hypertension, gingiva bleeding, diarrhea, fatigue, dysphonia, HFSR, and anorexia were likely caused by lenvatinib (2, 17, 34, 35). TACE is mostly related to elevated liver enzymes and post-embolism syndrome. However, there were no significant differences between groups in any parameter and they were manageable (2, 17, 34, 35).

In summary, there was no difference in the ORR between the lenvatinib plus TACE and lenvatinib monotherapy, according to Ando et al. (17). One possible reasons was that all HCC lesions of the included patients could be controlled by lenvatinib. A multicenter retrospective study showed that only the type of TKI was associated with tumor response (36). However, the OS of combined therapy was significantly longer for intermediate-stage HCC (17). The alternative lenvatinib plus TACE therapy improved the overall prognosis of patients compared with lenvatinib monotherapy, possibly because liver function could be preserved. The same result for the median OS was confirmed by Shimose et al. (2). According to Chen et al., the lenvatinib plus TACE group had significantly better ORR and OS than the lenvatinib monotherapy group in the REFLECT study, in spite that all included patients had HCC with PVTT; however, a larger sample size is needed for additional validation (35). Furthermore, we confirmed the superiority of lenvatinib plus TACE over TACE monotherapy in regard to ORR, PFS, and OS for uHCC (34). In addition, lenvatinib plus TACE is tolerable. The lenvatinib plus TACE group also confirmed its superiority in the following aspects. Shimose et al. reported that TACE is helpful in prolonging the administration time of lenvatinib. The administration time of lenvatinib in the AT group and non AT group was 13.7 months and 8.6 months, respectively (2). According to Kawamura et al., patients who achieved the lenvatinib-TACE sequential therapy after progression during lenvatinib therapy exhibited better post-progression survival (PPS), regardless of the CT enhancement pattern, whereas the heterogeneous enhancement pattern with irregularly shaped ring structures was correlated with a poorer PPS (39). Receiving TACE immediately after lenvatinib treatment could be an intense physical burden for patients (2). The median interval between TACE treatments was 74.7 d and 60.0% patients received TACE more than twice in the TACE group. However, the median interval between TACE treatments was 103.3 d and only 40.0% patients received TACE more than twice in the TACE plus lenvatinib group. Thus, the TACE plus lenvatinib therapy could decrease the number of TACE sessions and extend the interval time, which could be conducive to maintaining liver function, according to Fu et al. (34). Additionally, lenvatinib-TACE sequential therapy achieved tumor control even if the dose of lenvatinib was reduced or the drug was suspended, and the subsequent TACE treatment achieved tumor shrinkage (17). After the lenvatinib-TACE sequence therapy, a 78‐year‐old and an 80‐ year‐old patient with HCC at BCLC stage C received hepatectomy, which showed coagulative necrosis of the entire HCC in one case and a small amount of surviving HCC cells in the other case (40).

However, according to Matsuda et al., the diameter of the hepatic artery after TKI treatment, such as lenvatinib or sorafenib, decreased significantly, which may be caused by the normalization of tumor blood vessels, which limited TACE treatment after TKI treatment, even if the TACE treatment did not cause any complications (41). This could be a technical limitation and according to Xue et al., the decrease of hepatic vessel diameter will strengthen the effect of embolization and improve the survival benefit (42).

Eight clinical trails have been registered in ClinicalTrials. gov website to study the effect of TACE plus lenvatinib on uHCC, including preoperative treatment, prevention of postoperative recurrence. The registration time of the experiments is from January 2019 to May 2022, and the expected completion time is from August 2022 to March 2027.Two of them are from United States, six of them are from China, and two of them are multicenter studies. Whether lenvatinib and TACE are applied simultaneously or sequentially is also an urgent problem to be solved.

## Therapeutic responses of lenvatinib plus TACE therapy versus sorafenib plus TACE therapy

Whether lenvatinib plus TACE has advantages over sorafenib plus TACE is the subject of this section. Three studies with a total of 292 people were included (42–44). Two of them were prospective studies that explored the effect of TACE plus lenvatinib or sorafenib for patients with uHCC with PVTT (43, 44) and one propensity score matching retrospective study that addressed TACE with drug-eluting beads plus lenvatinib vs. sorafenib for advanced HCC (42). The outcomes are shown in **Table 2**.

**Table 2:** Lenvatinib plus TACE therapy compared with sorafenib plus TACE therapy.

Author/Reference numbers	Year	Country	Treatment (number of patients)	Follow-up time	Median OS/OS rate	Median PFS/PFS rate	TTP	ORR	Main characteristics of patients
Xiaoyan Ding (43)	2021	China	Lenvatinib+TACE (32)/sorafinib+TACE (32)	16.1 months	14.5 vs. 10.8 months	NA	4.7 vs. 3.1 months	53.1% vs. 25.0%	Tumor size (cm) >7.0(78.1% vs. 71.2%); Child-Pugh grade B (6.7% vs. 5.0%); PVTT I/II (65.6%vs78.1%), III/IV (34.4% vs. 21.9%); Extrahepatic spread (40.6% vs. 28.1%); AFP≥400(50% vs. 56.2%); BCLC stage C (100%) ALBI grade 2-3 (62.5% vs. 65.6%)
Biao Yang (44)	2021	China	Lenvatinib + TACE (38)/sorafenib + TACE (38)	NA	18.97 vs. 10.77 months	10.6 and 5.4 months	NA	66.8% vs. 33.3%	Tumor size (cm) >7 (63.2% vs. 68.4%); Child-Pugh grade B (2.6% vs. 2.6%); PVTT type I (52.6% vs. 52.6%), II (28.9% vs. 34.2%), III/IV (18.4% vs. 13.2%); Extrahepatic spread (15.8% vs. 15.8%); AFP≥400(63.2% vs. 60.5%); BCLC stage C (100%); ECOG PS 2 (28.9% vs. 23.7%)
Miao Xue (42)	2021	China	Lenvatinib+TACE (50)/sorafenib+TACE(100)	NA	14.9 vs. 12.3 months	NA	8.4 vs. 6.0 months	64.0% vs. 33.3%	Tumor size (cm) >5 (74.0% vs. 81.0%); Child-Pugh grade B (18% vs. 16%); PVTT (72.0% vs. 81.0%); Extrahepatic spread (54.0% vs. 45.0%); BCLC stage C (100%)

OS, overall survive; PFS, progression-free survival; TTP, time to progression; ORR, objective response rate; NA, not available; BCLC, Barcelona clinic liver cancer; ALBI, albumin-bilirubin; TACE, transarterial chemoembolization; PVTT, portal vein tumor thrombus; AFP, alpha-fetoprotein; ECOG PS, Eastern Cooperative Oncology Group performance status.

### Objective response rate

The response assessments were reported based on mRECIST. The ORR (CR + PR) of the sorafenib plus TACE group vs. lenvatinib plus TACE group was reported in three articles by Ding et al., Yang et al., and Xue et al., as 25% vs. 53.1%,P =0.039; 33.3% vs. 66.8%, p = 0.037; and 33.3% vs. 64%, P=0.008, respectively (42–44). These results verified that the ORR with the TACE plus lenvatinib treatment was superior to that of the TACE plus sorafenib treatment.

### Progression free survival or time to progression and overall survival

The PFS of the lenvatinib plus TACE and sorafenib plus TACE therapy was reported by Yang et al. as 10.6 vs. 5.4 months, p = 0.002 (44). The TTP of lenvatinib plus TACE therapy and sorafenib plus TACE therapy was reported by Ding et al. and Xue et al. as 4.7 vs. 3.1 months, P = .029 and 8.4 vs. 6.0 months, P=0.023, respectively (42, 43). The OS of lenvatinib plus TACE therapy and sorafenib plus TACE therapy was reported by Ding et al., Yang et al., and Xue et al. as 14.5 vs. 10.8 months, P = 0.17; 18.97 vs. 10.77 months, p = 0.022; and 14.9 vs. 12.3, P=0.043, respectively (42–44).The studies by Yang et al. and Xue et al. reported significant differences in the OS and PFS between the two groups, as noted above, after propensity score matching (PSM) (42, 44). Although Ding et al. reported no significant difference in OS between the two groups, the median OS for patients with advanced HCC including main PVTT receiving lenvatinib plus TACE was 14.5 months, which was longer than 13.6 months in the REFLECT trial (43). According to Ding et al., reasons why OS was not significantly different between the two groups could have been that a high proportion of patients with HCC (40.6%) in the sorafenib plus TACE group were switched to lenvatinib, and there were no PSM and few samples (43).

Univariable and multivariable analyses showed that the TACE frequency < 3, ECOG < 2, and treatment method were significantly important factors for longer OS according to Yang et al. (44). Ding et al. also reported that TACE plus lenvatinib significantly improved the TTP and OS. It was reported that a maximum liver tumor >7 cm was a critically negative prognostic factor and patients with HCC who achieved an objective response had significantly improved TTP and OS as well. However, according to this study, no significant lengthening or shortening of OS or TTP by AFP level, ECOG PS, type of PVTT, or extrahepatic metastasis differed from that in previous studies (43). We require larger sample sizes and more sophisticated experimental designs to explore this problem. Subgroup analysis showed that OS and PFS were significantly prolonged in the TACE plus lenvatinib group in patients with HCC with PVTT, especially PVTT type I/II, according to Ding et al., Yang et al., and Xue et al. (42–44). A retrospective study found that lenvatinib monotherapy increased both median OS (not reached vs. 187 d, p=0.0040) and ORR (53.8% vs. 14.3%, p=0.0193) compared with sorafenib in patients with HCC and PVTT type II/III (45). This further demonstrated the superiority of lenvatinib plus TACE in the treatment of various types of PVTT. TACE frequency < 3, ECOG < 2, larger and multiple tumors, cases with extrahepatic metastasis, and a higher AFP level appeared to benefit more from TACE plus lenvatinib (43, 44). Furthermore, in patients with FGF21 amplification, median OS was longer in the lenvatinib plus TACE group (10.4 months) than the sorafenib plus TACE group (5.7 months) according to Xue et al. (42). This result was in accordance with Finn et al. wherein a higher baseline FGF21 was related to longer OS with lenvatinib than sorafenib (46).

### Change in liver function and adverse events

There are no treatment-related deaths reported in these three articles, and AEs could also be controlled through drug reduction, drug withdrawal, and symptomatic treatment. Higher incidences of proteinuria, ascites, hoarseness, elevated bilirubin, decreased albumin, and hypothyroidism were observed in the lenvatinib plus TACE group compared with the sorafenib plus TACE group (42, 43). Higher incidences of HFSR and rash were observed in the sorafenib plus TACE group (42–44).The structural characteristics and different drug targets of sorafenib and lenvatinib played an important role (42). However, lenvatinib caused more AEs and a lower transition rate to second-line TKIs compared to sorafenib (2). A significantly higher incidence of ascites, decreased albumin, and elevated bilirubin suggested that lenvatinib has greater hepatic toxicity (43). According to Ding et al., lenvatinib plus TACE was tolerated in patients with HCC with the Child-Pugh classes A or B ≤7 (43).

The above results confirm the superiority of lenvatinib plus TACE over sorafenib plus TACE in terms of PFS, OS, and ORR. Lenvatinib led to greater AEs and hepatotoxicity. However, the lenvatinib plus TACE treatment is generally tolerable. Additionally, digital subtraction angiography (DSA) imaging authenticated that lenvatinib has a greater effect on vasoconstriction compared to sorafenib, which indicated that subsequent TACE treatment has a better embolic effect (42).According to a multicenter cohort study by Shimose et al., the median PFS time was 5.8, 3.2, and 2.4 months in the lenvatinib, sorafenib, and TACE groups in patients with intermediate-stage HCC refractory to TACE, respectively (47).Ding et al. also identified the use of the camrelizumab (a kind of ICI) after disease progression as a positive predictive factor for survival (43). This leads to the next topic to be discussed, the suitability and efficacy of lenvatinib plus TACE combined with immunotherapy.

## Therapeutic responses of lenvatinib plus TACE with PD-1 inhibitors

In the previous section, we explained the advantages of lenvatinib plus TACE over TACE alone in patients with uHCC, lenvatinib monotherapy in patients with intermediate-stage HCC, and sorafenib plus TACE in patients with advanced-stage HCC, especially with PVTT. However, these advantages were inconspicuous and the most research was imperfect. Whether lenvatinib plus TACE and PD-1 inhibitors can play a greater role is discussed in this section. Our review embodied 6 studies on lenvatinib plus TACE and PD-1 inhibitors in a total of 465 people with uHCC, of which only 2 studies were randomized and controlled, and the other four were single arm studies, indicating the effectiveness and safety of the triple therapy (31–33, 48–50). All of the articles here were from China, and the PD-1 inhibitors are developed in China: toripalimab,camrelizumab,pembrolizumab,sintilimab,tislelizumab (31–33, 48–50). The outcomes are shown in **Table 3**. Immunotherapy had a longer onset period than TACE, but lasted longer (51).

**Table 3:** Lenvatinib plus TACE combined with PD-1 inhibitors.

Author/Reference numbers	Year	Country	Treatment (number of patients)	Follow-up time	Median OS/OS rate	Median PFS/PFS rate	TTP	ORR	Main characteristics of patients
Mingyue Cai (32)	2022	China	Lenvatinib+TACE+PD-1 inhibitors(sintilimab/tislelizumab/camrelizumab) (41) vs lenvatinib+TACE (40)	13.7 months	16.9 vs. 12.1months.	7.3 vs. 4.0 months	NA	56.1% vs.32.5%	Tumor size (cm) 12.3 ± 4.8/13.6 ± 5.1; Child-Pugh grade B (9.8% vs. 17.5%); PVTT type III (36.6% vs. 45.0%); Extrahepatic spread (41.5% vs. 47.5%); AFP≥400(51.2% vs. 55.0%); BCLC stage C (100%)
Song Chen (48)	2021	China	Lenvatinib+TACE+ PD-1 inhibitors (pembrolizumab) (n=70) vs. lenvatinib+TACE (n=72)	27 months	18.1 vs. 14.1 months	9.2 vs. 5.5 months	NA	47.1% vs. 27.8%	Brain metastasis (68.6% vs.72.2%); AFP≥400(64.3% vs. 61.1%); BCLC stage B (67.1% vs.62.5%), C (32.9% vs. 37.5%) ALBI grade 2 (65.7% vs. 70.8%)
Ying Teng (33)	2021	China	Lenvatinib+TACE+PD-1 inhibitors(sintilimab/camrelizumab)(53)	15.4 months	not reached	8.5 months	NA	54.9%	Child-Pugh grade B (35.8%); Vascular invasion (47.2%); Extrahepatic spread (79.2%); AFP≥400(34.0%); BCLC stage B (43.4%), C (56.6%)
Juanfang Liu (49)	2021	China	Lenvatinib+TACE+PD-1 inhibitors (camrelizumab) (22)	NA	24 months	11.4 months	NA	NA	Tumor burden >50% (36.4%); Child-Pugh grade B (27.3%); PVTT (50.0%); AFP≥400(68.2%); BCLC stage B (54.5%)/C (45.5%); ECOG PS 2 (36.4%)
Fei Cao (50)	2021	China	Lenvatinib+TACE+PD-1 inhibitors (sintilimab) (52)	12.5 months	23.6 months	13.3 months	NA	46.7%	Child-Pugh grade B (11.5%); Macroscopic vascular invasion (36.5%); Extrahepatic spread (40.4%); AFP≥400 (34.6%); BCLC stage B (25.0%)/C (75.0%); ALBI grade 2-3 (80.8%)
JiaYi Wu (31)	2021	China	Lenvatinib+TACE+PD-1 inhibitors(sintilimab/tislelizumab/camrelizumab/toripalimab/pembrolizumab)(62)	12.2 months	not reached	not reached	NA	Investigator and BICR-assessed ORR were 80.6% and 77.4%	Tumor size (cm) ≥10 (50%); Child-Pugh grade B (6.7% vs. 5.0%); PVTT type I (6.5%), II (19.4%), III/IV(17.7%); Extrahepatic spread (9.7%); AFP≥400(51.6%); BCLC stage A (9.7%)/B (33.9%)/C (56.5%)

OS, overall survive; PFS, progression-free survival; TTP, time to progression; ORR, objective response rate; PD-1, programmed death factor 1; NA, not available; BCLC, Barcelona clinic liver cancer; ALBI, albumin-bilirubin; TACE, transarterial chemoembolization; PVTT, portal vein tumor thrombus; AFP, alpha-fetoprotein; ECOG PS, Eastern Cooperative Oncology Group performance status; BICR, blinded independent central review.

### Objective response rate

The reported tumor responses were assessed by mRECIST. The ORR (CR + PR) of the lenvatinib plus TACE and PD-1 inhibitors group vs. the lenvatinib plus TACE group was reported by Chen et al. and Cai et al., being 47.1% vs. 27.8%, p=0.017; 56.1% vs. 32.5%, P=0.033, respectively (32, 48). The ORR in the remaining three articles was 54.9%, 46.7%, as well as 80.6% assessed by an investigator and 77.4% assessed by a blinded independent central reviewer (BICR) (31, 33, 50).

### Progression free survival and overall survival

The PFS of the lenvatinib plus TACE and PD-1 inhibitors group vs. the lenvatinib plus TACE group was reported as 9.2 vs. 5.5 months, p=0.006 and 7.3 vs. 4.0 months, P=0.002, respectively (32, 48). The PFS of the remaining three articles was 8.5, 11.4, and 13.3 months (33, 49, 50).The median OS of the lenvatinib plus TACE and PD-1 group vs. the lenvatinib plus TACE group was reported as 18.1 vs. 14.1 months, p=0.004; 16.9 vs. 12.1 months, P=0.009, respectively (32, 48). The median OS of the remaining two studies was 24 months and 23.6 months reported by Liu et al., Cao et al. (49, 50).

### Change of liver function and adverse events

According to Teng et al., 3.8% patients with HCC experienced upper gastrointestinal bleeding and died; however, it is unknow if this was related to treatment (33). Furthermore, this occurred in 7% patients in the IMbrave 150 trial (24). Cao et al. reported that a total of 3.8% of patients with HCC experienced grade 5 AEs, including one developed abnormal liver function, upper gastrointestinal bleeding and death on day 134 (50).Significant differences occurred in terms of hypertension, nausea, and rash in the lenvatinib plus TACE and pembrolizumab group vs. in the lenvatinib plus TACE group according to Chen et al. (48). Liu et al. reported that 1 week after the triple therapy, the levels of aspartate aminotransferase (AST) and alanine aminotransferase (ALT) were elevated, However, there was no significant change in total bilirubin. All of these levels returned to baseline 1 month after the triple therapy (49).

In summary, despite having a higher tumor burden, higher level of AFP, larger proportion of patients with Child-Puge grade B, ECOG PS 2, PVTT, and extrahepatic metastasis, the studies confirmed superior PFS, OS, or ORR compared with Phase Ib Study and the IMbrave150 trial (31, 49, 50). According to Cai et al. and Chen et al., lenvatinib plus TACE and PD-1 inhibitors therapy had advantages in ORR, PFS, and OS compared with lenvatinib plus TACE therapy. However, the PFS and OS in the triple therapy group in these two studies were relatively short. A considerable proportion of patients with HCC and extrahepatic metastasis (41.5% and 68.6%), AFP levels ≥400ng/ml (51.2% and 64.3%) could have been the reasons (32, 48). Furthermore, a significant proportion of patients have main PVTT (36.6%), the heavy tumor burden (largest tumor size of 12.3 ± 4.8 cm) could also lead to the limited survival benefit of the triple therapy, according to Cai et al. (32). Despite improvements in ORR compared with the Phase Ib Study and the IMbrave150 trial, according to Teng et al., the median PFS and OS was shorter than that of lenvatinib plus pembrolizumab (33, 49, 50, 52). The reasons could include the high proportion of patients with HCC with TACE failure, previous TKI treatment failure (45.3%), and inadequate follow-up. The median PFS was 11.2 months for patients with HCC after first-line treatment with PD-1 inhibitors, which was longer than that of second-line therapy (6.2 months) and that of PFS reported by Phase Ib Study which suggested that the triple therapy should be used in patients with HCC as early as possible (33, 49, 50). Lenvatinib after failure of PD-1 inhibitors was longer than that of lenvatinib as the first-line therapy. The effect of PD-1 inhibitors binding to CD8+T cells being sustained for more than several months might be one of the reasons (53). However, different results were reported by Yao et al., in which changes in signaling pathway, epigenetics, and the upregulation of other checkpoints (such as T-cell immunoglobulin and mucin domain 3 (TIM3) and cytotoxic T-lymphocyte-associated protein 4 (CTLA-4)), the history of PD-1 inhibitors resulted in a poorer PFS (36). This is another problem that needs to be addressed in the future. Additionally, a study for patients with uHCC with 69.6% in BCLC stage C, macroscopic vascular invasion (33.9%) and extrahepatic metastasis (51.8%) achieved an ORR of 67.9%, a median PFS of 11.9 months, and a median OS of 23.9 months in the triple therapy group (54). Xiang et al. reported that the triple therapy for patients with intermediate-stage HCC had an ORR of 64.3%, median OS of 26.0 months, and median PFS of 22.5 months (55). In addition, a global randomized Phase 3 LEAP-012 Study conducted in the United States is ongoing to compare TACE with or without lenvatinib plus pembrolizumab for intermediate-stage HCC that are not amenable to curative treatment. The research plans to include 950 patients from different countries and was expected to be completed in 2029.Ten clinical trails have also been registered in ClinicalTrials. gov website to study the effect of TACE plus lenvatinib and ICIs on uHCC, including conversion therapy. The registration time of the experiments were from May 2020 to May 2022, and the expected completion time is from December 2022 to January 2027.Nine experiments are from China and one is from the United States. ICIs planned to be used include sintilimab, tislelizumab, camrelizumab, toripalimab, pembrolizumab, envafolimab, tremelimumab and durvalumab. The choice of ICIs is a problem, the application sequence of triple therapy is also urgent problems to be solved.

PVTT and extrahepatic metastasis indicated a lower OS in patients with advanced HCC and extrahepatic metastasis indicated a shorter PFS (49). Cai et al. also reported similar results, wherein the main PVTT, extrahepatic metastasis, and treatment options were identified as the independent prognostic factors for OS. Treatment option and extrahepatic metastasis were identified as the independent prognostic factors for PFS (32). Combined positive score (CPS)>1 indicated PD-L1 positivity (56). Chen et al. reported that a higher PD-L1 CPS was associated with a longer OS with anti-PD-L1 treatment (48). In addition, a high conversion rate was related to PD-L1 positive expression (57, 58). However, PD-L1 expression was related to tumor aggressiveness based on tumor resection specimens (59). OS and ORR in patients with HCC treated with nivolumab affected the expression of tumor PD-1 and PD-L1 at baseline (60). Subgroup analyses indicated that the triple therapy might be better employed for patients with HCC before the main PVTT and having a tumor number >3 or extrahepatic metastasis. The reasons could be that TACE acted on intrahepatic tumors rather than extrahepatic metastases and the effect of TACE on multiple tumors was constrained (32). Immune evasion in extrahepatic tumors could be another reason (34). According to Chen et al., a distinct, durable response was observed in patients with HCC with intrahepatic tumors who recieved lenvatinib plus TACE, suggesting that lenvatinib plus TACE therapy has a short-term anticancer effect for these patients, but the effect on patients with distant metastasis was limited (48). The higher the ORR of overall tumor (56.1% vs. 32.5%, P=0.033) and intrahepatic tumor (65.9% vs. 37.5%, P=0.011) in the lenvatinib plus TACE and PD-1 inhibitor group and lenvatinib plus TACE group was reported by Cai et al., which indicated that PD-1 inhibitors improved ORR than lenvatinib plus TACE therapy, both for the intrahepatic tumor and overall tumor (32). Wu et al. reported an ORR of 66.7% at BCLC stage A, 76.2% at BCLC stage B, and 80% at BCLC stage C. ORRs were not different at various BCLC stages (31).

Chen et al. reported a tumor reduction rate of 90.0% in the lenvatinib plus TACE and pembrolizumab group vs. 72.2% in the lenvatinib plus TACE group, *p*=0.007 (48). The study of Wu et al. showed a tumor reduction rate was 91.9% in the lenvatinib plus TACE and PD-1 inhibitor group (31). The rate of conversion therapy in Chen et al. was 25.7% vs. 11.1%, p=0.025. Among patients undergoing surgery, 22.2% died in the triple therapy group and 75.0% died in the duplex group, *p*=0.012 (48). These data corroborated that triple therapy hindered the progression of uHCC more compared than the duplex therapy (48). Wu et al. reported that a total of 33 patients with HCC reached the resectable standard (3 with BCLC stage A, 11 with BCLC stage B, and 19 with BCLC stage C). Twenty-nine patients underwent resection (31). Pathological CR and major pathological response (no active tumor cells were found in the resected specimens, and less than or equal to 10%, respectively) were observed in 16 and 24 patients, respectively (31). The 5-year survival rate of patients who underwent surgical resection after downstaging conversion therapy was similar to that of patients who underwent surgical resection at the beginning (48).

## Why the combination therapy of lenvatinib plus TACE with PD-1 inhibitors is promising

### Combination of TACE and lenvatinib

TACE only worked on intrahepatic lesions, and had no effect on extrahepatic metastasis; thus, combination with systemic therapy is necessary (61). In addition, TACE could lead to necrosis of tumor tissue and upregulate the expression of hypoxia-inducible factor 1-α (HIF-1α), vascular endothelial growth factor (VEGF), and fibroblast growth factor (FGF), which could stimulate tumor recurrence or growth (2, 34, 42, 61). However, lenvatinib administration after TACE could suppress the effects of angiogenic factors (2, 40). Lenvatinib pretreatment could promote the normalization of tumor feeding arteries, lessen the pressure of intertumoral interstitial, and reduce vascular permeability. This change could improve the distribution of lipiodol and drug loaded microspheres mixed with chemotherapy drugs, to enhance the therapeutic effects of TACE (26, 40). Additionally, the shrinkage and reduction of tumor feeding arteries led to the reduction of the embolic material and lipiodol dose, which could be helpful in maintaining liver function. As compared with lenvatinib, TACE has been reported to worsen the hepatic functional reserve (40). However, not all lesions responded to lenvatinib because of the high heterogeneity of the HCC. Tumor progression after lenvantinib therapy, and second-line drugs were used. However, if TACE could control these “no response” lesions, lenvatinib could continue to be used. Compared with other drugs, lenvatinib had a higher tumor response rate (2, 17, 62). Thus, the purpose of lenvantinib plus TACE therapy is to provide a continuous deep response without deterioration of liver function, improve the prognosis of patients with intermediate-stage hepatocellular HCC, and prolong the time of transformation to advanced HCC (17).

### Compared with sorafenib, lenvatinib had advantages in combining with TACE

The possible mechanisms of the different effects of lenvatinib and sorafenib are described next. First, the two drugs have different targets., lenvatinib acted as an inhibitor of VEGF receptors (VEGFR)1–3, FGF receptors (FGFR) 1–4, platelet-derived growth factor receptor (PDGFR) -α, proto-oncogenes KIT, and RET (8, 63). Sorafenib primarily suppressed the function of Raf kinase, VEGF, and PDGF (64, 65). The FGF/FGFR signaling pathway led to the activation of multiple downstream pathways, such as Ras/MAPK and PI3K/AKT signaling pathways, which promoted cell proliferation and angiogenesis. Abnormal expression of FGF19/FGFR4 accelerated HCC progression (63). In addition to the VEGF/VEGFR signaling pathway, the FGF/FGFR signaling pathway also works on tumor progression in HCC. The dual inhibition of lenvatinib on VEGFR and FGFR signaling pathways enhanced its antitumor effect in HCC (42, 63). Furthermore, according to previous studies, FGF 19, 21, and 23 could be tied to OS in patients with uHCC treated with lenvatinib or sorafenib (42, 63, 64, 66, 67). The development of HCC is promoted by FGF21 amplification *via* the TGF-β signaling pathway and patients having higher baseline FGF21 appeared to have better OS with lenvatinib than sorafenib (42).Cell survival, growth, proliferation, and differentiation was also limited by lenvatinib *via* blocking the RET receptor, which is associated with numerous signaling pathways, including PI3K/AKT and RAS/MAPK pathways (42, 63). Second, the two drugs also bind to target kinases in different ways: the binding mode of lenvatinib to VEGFR2 is Type V, and of sorafenib is Type II. The former binding mode is more closely related to VEGFR2 (67). VEGFR2 has a high-affinity with VEGF on vascular endothelial cells and HCC cells. The binding of VEGFA and VEGFR2 causes activation of the phospholipase-Cγ (PLCγ), Ras/MAPK, and PI3K/AKT signaling pathways. These signaling pathways are involved in the proliferation of tumor cells, endothelial cells, and an increase in vascular permeability (63). Lenvatinib significantly reduced the tumor microvessel density in HCC by blocking VEGFR and had a stronger effect than sorafenib in various preclinical models (63). Finally, the immunomodulatory activity of lenvatinib targeting FGFR has been demonstrated in recent studies. There were no differences in antitumor activity between lenvatinib and sorafenib in immunodeficient mice, although lenvatinib was confirmed to have additional antitumor activity in immunocompetent mice (68).

### Immune response activated by TACE

According to Montasser et al., tumor specimens from patients with TACE therapy showed substantially higher PD-L1 expression in cells. PD-1 and PD-L1 expression in inflammatory cells were higher in TACE-resected tumors than non-TACE group (29). According to this report, TACE therapy was related to the increase of PD-1 and PD-L1 expression in HCC, and could be a promising therapeutic option in combination with immunotherapy (29). Chen et al. reported that a higher PD-L1 CPS was associated with a longer OS with anti-PD-L1 treatment (48). A high conversion rate was related to PD-L1 positive expression in the previous studies (57, 58). Membranous PD-1/PD-L1 (mPD-1/mPD-L1) and soluble PD-1/PD-L1 (sPD-1/sPD-L1) are the two forms of the PD-1/PD-L1 molecules. SPD-L1 is mainly separated from mPD-L1, partly reflects the level of mPD-L1, and was easy to measure. Studies have found that the expression of PD-L1 was related to tumor staging, prognosis, and could be potential biomarker of the onset, development, and prognosis of HCC guiding to immunotherapy. SPD-L1 level was higher in patients with BCLC stage C, PVTT, or beyond the up-to-seven criterion. According to Ma et al., the level of sPD-L1 in CR patients receiving TACE therapy was lower than that of PR and SD patients, which further confirmed that the level of sPD-L1 was related to the prognosis and responsiveness to treatment of patients (69). Tumor apoptosis or necrosis caused by TACE promoted the release of chemokines and inflammatory mediators, which increased the level of sPD-L1 (29, 69). When the tumor burden was reduced by TACE, the level of sPD-L1 decreased (69). Approximately 1 week after TACE therapy, the immune inhibition becomes increasingly dominant in TME, because sPD-L1 continues to increase. This period is the best time to apply ICIs and fully activate the immune system for the eradication of tumor cells (69, 70).

Additionally, TACE was reported to promote T-cell activation. Tumor cell necrosis caused by TACE increased the release of tumor-associated antigens, recruited DCs and increased CD4+T cells (57). According to Ren et al., 1 to 5 weeks after TACE, the proportion of Treg cells was significantly lower than before TACE, and this result indicated that a positive regulatory effect on immune function should occur after TACE. This study also showed that the proportion of CD4+T cells and the ratio of CD4+/CD8+T cells prominently increased in HCC from 1 to 5 weeks after TACE, CD8+T cells slightly increased; however, there was no statistical significance and these data confirmed that the immune function was restored in HCC after TACE (71). The increase of CD4+ and CD8+ cells after TACE has also been reported in previous studies and it was related to a better response to TACE therapy (69). The above confirmed the improvement of immune function within 1 month after TACE (71). Yang et al. reported that CD4+T cells and the ratio of CD4+/CD8+T cells decreased in 1 month after lenvatinib plus TACE and PD-1 inhibitor therapy. Nevertheless, CD8+ T, CD3+ T, and NK cells increased from 1 to 4 months. In general, triple therapy could activate immune function and maintained it for a long time (6). However, hypoxia and overexpression of VEGF as a result of TACE led to an immunosuppressive TME by increasing Treg cells, myeloid-derived suppressor cells (MDSCs), and mast cells, recruiting monocytes from bone marrow, and raising tumor-infiltrating macrophages. Moreover, VEGF inhibited the development of T cells and the maturation of DCs (50, 58, 66). VEGF was also reported to modulate the checkpoint expression of CD8+T cells in HCC (72). The expression of PD-1 increased in peripheral mononuclear cells (6, 7). ICIs activate interferon-γ (IFN-γ)+ Type 1 T helper (Th1) cells to normalize the tumor vasculars and improve hypoxic environments (6). TACE synergized with PD-1 inhibitors and increased T lymphocytes (57). The immune response induced by TACE is complex, but there was indeed a synergistic effect with PD-1 inhibitors.

### Studies on the mechanisms of lenvatinib combined with PD-1 inhibitors therapy

In addition to the role of TACE, the oxygen content of tumor cells was dramatically lower than that of normal liver cells, which increased the angiogenic growth factors, including VEGF and FGF and led to immune disorders (73). The expression of PD-1, CTLA-4, and Tim-3 were upregulated by FGF and VEGF on T cells. TIM-3 also promoted the exhaustion of T cells (70). When FGF and VEGF were combined, these effects were strengthened (30). After PD-1 inhibitors therapy, the expression of VEGF and FGF in patients with PD was significantly higher than that of SD patients (30). Lenvatinib inhibited these angiogenic growth factors and was associated with T-cell activation, enhanced the antitumor immunity, and increased the efficacy of PD-1 inhibitors (54, 55, 74). Additionally, the JAK/STAT3 signaling pathway was activated by FGFR2 signals accompanied with increasing PD-L1 expression according to Li et al. FGFR2 was inhibited by lenvatinib (75). Yi et al. showed that PD-1 inhibitors increased the level of interleukin 2 (IL-2); nevertheless, lenvatinib inhibited IL-2-mediated Treg differentiation by targeting FGFR4 and restrained STAT5 phosphorylation. Lenvatinib and FGFR4 knockdown lead to the activation of GSK3β, which destabilized PD-L1 *via* proteasome degradation (76). Lenvatinib decreased the expression of PD-L1 on human umbilical vein endothelial cells (HUVEC). However, it did not affect the expression of PD-L1 on tumor cells and restored T-cell function and retained the sensitivity of tumor cells to PD-1inhibitors (30). Adachi et al. reported that the activation of FGFR inhibited the IFN-γ-signaling pathways in mouse and human renal cell carcinoma (RCC) cell lines (66). IFN-γ could enhance the immune response by recruiting other leukocytes (76). The IFN-γ signaling pathway also facilitated tumor recognition by cytotoxic CD8+T cells, increased tumor immunogenicity, and caused rejection of the tumor by the host immune system. An active IFN-γ-signaling pathway enhances antitumor activity of lenvatinib plus PD-1 inhibitors. IFN-γ also activated the JAK/STAT1 signaling pathway and increased its target genes, including PD-L1. Lenvatinib blocked FGFR, which also led to an increase in PD-L1 (66). The increased PD-L1-positive area after PD-1 inhibitor monotherapy further extended after lenvatinib plus PD-1 inhibitors. Adachi et al. held that the increase of PD-L1 will enhance the effect of PD-1 inhibitors (66). Lenvatinib also increased neutrophil and upregulated PD-L1 expression on neutrophils in the TME (77). In conclusion, the effect of lenvatinib on PD-L1 expression remains controversial and has not been finalized. Koganemaru et al. reported that PD‐L1 overexpression on tissue-infiltrating mononuclear cells was related to a good prognosis yet poor prognosis of tumor cells (78). PD-L1 overexpression on tumor cells or inflammatory cells had a considerable relationship with tumor aggressiveness, such as poor differentiation, high AFP levels, satellite nodules, and vascular invasion. However, PD-L1 expression was thought to represent a biomarker predictive of drug sensitivity (59). Although according to the current study, PD-L1 expression was closely related to a poor prognosis, it also promoted antitumor activity of lenvatinib plus PD-1 inhibitor therapy according to Adachi et al. (66). The role of PD-L1 in the prognosis of HCC and in the prediction of lenvatinib treatment effect needs further verification, but the superiority of lenvatinib combined with PD-1 inhibitors should not be ignored. In addition to the above mechanisms, lenvatinib also enhanced the efficacy of PD-1 inhibitors by increasing the proportion of activated CD8+T cells and secreting IFN-γ and granzyme B. IFNγ+ CD8+ T cells increased more in the combination therapy group. Moreover, in immunodeficient mice, the antitumor activity of lenvatinib decreased because of the absence of CD8+T cells. CD4+ T cells also increased with lenvatinib therapy. Furthermore, lenvatinib decreased monocytes, macrophages, and TAMs (30, 66, 68, 79). A low concentration of lenvatinib acted as an immunoregulator (30). Long-term immune memory was formed with lenvatinib plus PD-1 inhibitor therapy, the TME was modulated, and the cytotoxic effect of T cells enhanced (30). Lenvatinib plus PD-1 inhibitor therapy also reduced tumor vessel density (30, 42).

According to the article published by Yang et al., CD8+ T cells increased significantly after 1 month of the TKIs plus TACE and inhibitor therapy, which reflects the activation of cellular immunity. However, CD8+T cells were relatively stable after PD-1 inhibitor monotherapy or TACE therapy alone (80). A significant increase in circulating CD8+ T cells was observed until 3 months after tremelimumab plus TACE therapy (81). Patients receiving PD-1 inhibitors-based combination immunotherapy, appeared to experience a decrease in B cells accompanied by an increase in Ig G, kappa light chains (Ig к), and lambda light chains (Ig λ). B cells migrated from the bone marrow to the secondary lymphoid organs, activated by antigens and underwent isotype switching to Ig G. Therefore, the reduction in circulating B cells likely occurred because of the isotype switch to antibodies, indicating that humoral immunity plays an important role in TKIs plus TACE and inhibitors therapy (6). Additionally, Ig G, Ig κ, and Igλ increased at the time of response, and decreased to the baseline with tumor progression. CD8+ T and B cells did not show this trend. Therefore, circulating Ig G, Ig κ, and Igλ could serve as potential biomarkers (6).

## Conclusions

The advantages of lenvatinib plus TACE over lenvatinib monotherapy in patients with HCC in intermediate stage, especially beyond the up-to-seven criterion, over TACE in patients with uHCC, and over sorfenib plus TACE in patients with advanced-stage HCC, especially with PVTT, are described in detail in this review. Lenvatinib plus TACE therapy is preferable in patients with HCC with high tumor burden, poor liver function, and numerous heterogeneous lesions in the intermediate stage and has advantages over sorafenib plus TACE in patients with PVTT. Lenvatinib plus TACE and PD-1 inhibitors therapy improved OS, PFS, and ORR compared with lenvatinib plus TACE therapy, and is a promising treatment for patients with uHCC at various BCLC stages.

## Author contributions

FC and LS proposed ideas and completed outline of this review. FM, XX and QL found out the relevant literatures and carried out data collection. HW and XL performed data analysis. LS wrote the original draft and edited the manuscript. GL and FC supervised. All authors contributed to the article and approved the submitted version.

## Funding

This study was funded by the Natural Science Foundation of Shandong Province (grant no. ZR2019BH041 to FC), the Nature Science Foundation of China (grant no. 81803008 to FC) and the cultivating fund of the First hospital of Shandong First Medical University (grant no. QYPY2021NSFC0616 to FC).

## Acknowledgments

We would like to thank Editage (www.editage.cn) for English language editing.

## Conflict of interest

The authors declare that the research was conducted in the absence of any commercial or financial relationships that could be construed as a potential conflict of interest.

## Publisher’s note

All claims expressed in this article are solely those of the authors and do not necessarily represent those of their affiliated organizations, or those of the publisher, the editors and the reviewers. Any product that may be evaluated in this article, or claim that may be made by its manufacturer, is not guaranteed or endorsed by the publisher.
